# Advanced synergistic antibacterial interfaces enabled by micro/nanostructure–superhydrophobicity coupling: mechanisms and applications

**DOI:** 10.3389/fmicb.2026.1928666

**Published:** 2026-08-27

**Authors:** Qiuxu Guo, Yongfeng Wang, Jiayu Wang, Linxue Wang, Lei Wang

**Affiliations:** Department of Pediatric Outpatient, The First Hospital of Jilin University, Changchun, China

**Keywords:** bio-inspired surfaces, mechanisms, micro/nanostructures, superhydrophobicity, synergistic antibacterial surfaces

## Abstract

Bacterial contamination poses serious threats to human health and material integrity in fields such as healthcare, food safety, and marine engineering. Conventional chemical antibacterial strategies increasingly fail to meet practical demands due to the emergence of bacterial drug resistance and concerns over environmental sustainability. Inspired by functional biological surfaces, physical antibacterial strategies based on the synergistic regulation of micro/nanostructures and superhydrophobicity have attracted growing attention. However, the coupling relationships among microstructural parameters, wetting states, interfacial mechanical behaviors, and bacterial responses remain insufficiently understood. This review systematically summarizes the bactericidal mechanism induced by micro/nanostructures and the anti-adhesive mechanism induced by superhydrophobicity. Furthermore, the representative application scenarios in biomedical devices, food processing, and marine environments are discussed. Finally, current progress, key challenges, and future perspectives in the development of micro/nanostructured superhydrophobic synergistic antibacterial surfaces are critically reviewed. Overall, this strategy offers significant potential for environmentally friendly and long-lasting antibacterial solutions. However, a deeper understanding of the coupling mechanisms among surface structure, wettability, and cellular responses is still required to fully exploit its antibacterial capabilities.

## 1 Introduction

Bacterial contamination is prevalent in a wide range of fields, including medical devices (Chen et al., 2026; Bouhrour et al., 2024), food processing (Aung and Kim, 2024; Carrascosa et al., 2021; Nogueira et al., 2025), marine engineering (Tuck et al., 2022), and public infrastructure (Li et al., 2023). It can readily lead to problems such as cross-infection, material corrosion, and biofouling, thereby posing serious threats to human health and causing substantial economic losses. The process of bacterial contamination typically involves two sequential stages (Lichter et al., 2009). Stage I corresponds to the initial interaction between bacteria and the material surface. Stage II involves both specific and non-specific interactions between proteins on bacterial surface appendages (such as fimbriae or pili) and binding molecules on the material surface, ultimately leading to the formation of mature biofilms. Traditional antibacterial strategies primarily rely on chemical disinfectants or antibiotics. However, their long-term and excessive use not only accelerates the emergence of bacterial resistance but also raises concerns regarding cumulative environmental pollution (Sousa et al., 2025). Consequently, antibacterial approaches based on material surface functionalization have attracted increasing attention in recent years. In particular, bio-inspired strategies that mimic natural micro/nanostructures to achieve physical bactericidal effects and bacterial adhesion inhibition have emerged as promising alternatives (Priya et al., 2026; Cao et al., 2024; Linklater et al., 2021; Xia X. H. et al., 2025).

Numerous natural surfaces exhibit typical synergistic antifouling and antibacterial characteristics derived from the coupling of surface structure and wettability. For example, the superhydrophobic micro/nano-hierarchical structures of lotus leaves significantly reduce droplet adhesion, while the highly ordered nanocolumn arrays on the wings of cicadas and dragonflies can mechanically penetrate bacterial cell membranes upon contact, thereby actively killing bacteria (Dou et al., 2025). Such multiscale structures enable the regulation of surface wettability, reduction of interfacial free energy, and modulation of bacterial–surface interactions, providing important inspiration for the antibacterial design of artificial surfaces (Manivasagam et al., 2022). Motivated by these natural prototypes, researchers have begun to fabricate artificial antibacterial surfaces with tailored wettability and physical bactericidal functionality through strategies including microstructure architecture design, surface energy modulation, mechanical response engineering, and multiphysical field coupling.

A growing number of review articles have systematically reported the fabrication methods, structural design and antibacterial applications of functional antibacterial materials (Larranaga-Altuna et al., 2021; Zhou et al., 2023). Most existing reviews mainly focus on the classification of antibacterial strategies, summary of preparation technologies, or macroscopic application progress of superhydrophobic antibacterial surfaces. However, they generally overlook the intrinsic coupling mechanism between multiscale microstructure characteristics and superhydrophobic antibacterial performance, and fail to elaborate on the quantitative correlation between structural parameters, interfacial wetting behavior and bacterial inactivation responses. Current studies further indicate that relying solely on a single surface structure or surface chemical modification often fails to simultaneously achieve long-term anti-adhesion performance and highly efficient bactericidal activity under prolonged service conditions (Chug and Brisbois, 2022; Jia et al., 2023; Zhong et al., 2025). Consequently, the synergistic “micro/nanostructure–superhydrophobicity” design paradigm has gradually emerged as a central strategy in antibacterial material engineering. By constructing well-defined micro- and nanoscale geometric architectures, surface wettability can be effectively tuned toward superhydrophobic state, thereby suppressing the initial adhesion of bacteria. Simultaneously, structure-induced mechanisms, such as cellular stretching, shearing, membrane puncturing, and interfacial energy-driven effects, can be exploited to realize physical bactericidal functions (Baulin et al., 2025; Larranaga-Altuna et al., 2021; Zhou et al., 2023; Chen et al., 2023a). Notably, this synergistic mechanism exhibits distinct advantages, including the absence of external chemical agents, negligible risk of inducing bacterial resistance, and long-term operational stability, highlighting its great potential for next-generation green antibacterial technologies.

In recent years, rapid advances in micro-/nano-fabrication techniques, such as femtosecond and nanosecond laser processing, photolithography, electrochemical methods, three-dimensional printing, and ion beam etching, have enabled the precise construction of controllable multiscale surface architectures and the programmable regulation of surface wettability (Sun et al., 2026; Cai et al., 2022; Lin and Hong, 2021; Basu et al., 2025; Lian et al., 2024; Seoane-Viano et al., 2021; Yang J. M. et al., 2025; He S. K. et al., 2025). Meanwhile, emerging concepts such as underwater superhydrophobicity (Mehanna et al., 2021; Fauconnier et al., 2025; Wu et al., 2023) have continuously expanded the design space of antibacterial surfaces, driving their evolution from single-function systems toward multifunctional and synergistic platforms. Despite these advances, the coupling relationships among microstructural parameters, wetting states, interfacial mechanical behaviors, and bacterial responses remain insufficiently understood. Furthermore, challenges related to structural manufacturability, durability, and the long-term stability of antifouling and antibacterial performance under complex service environments remain critical issues that must be urgently addressed. The above research gaps restrict the in-depth development and engineering application of high-performance synergistic antibacterial surfaces, making it necessary to conduct a targeted and systematic summary.

Different from previously published reviews that focus on macroscopic technology induction and application overview, this paper emphatically summarizes the latest research progress of micro/nanostructured superhydrophobic synergistic antibacterial surfaces from the perspective of interfacial synergistic mechanism and structural parameter regulation. Particular emphasis is placed on structure-driven mechanical bactericidal mechanisms and interfacial behaviors under superhydrophobic state, as well as their synergistic effects. In addition, the representative application fields of micro/nanostructured superhydrophobic synergistic antibacterial surfaces are discussed. Finally, current research challenges and future development directions are outlined, aiming to provide a theoretical foundation and technical guidance for the design of efficient, durable, and environmentally friendly antibacterial surfaces.

## 2 Individual antibacterial mechanisms of micro/nanostructures and superhydrophobicity

To standardize the nomenclature and eliminate conceptual ambiguity throughout this manuscript, four core functional terms are explicitly defined and distinguished herein: antifouling refers to the capability of inhibiting surface contaminant deposition and biofilm formation; anti-adhesion specifically describes the suppression of initial bacterial attachment on material surfaces, serving as a pivotal subset of antifouling effects; bactericidal corresponds to the active mechanical or chemical action that damages bacterial structures and inactivates viable bacteria; antibacterial is a generalized umbrella term that comprehensively covers the integrated effects of anti-adhesion, antifouling and bactericidal performance. Correspondingly, the air insulation effect of superhydrophobic surfaces dominates the anti-adhesion and antifouling functions, the mechanical piercing and stretching behaviors of micro/nanostructures realize bactericidal activity, and the complementary combination of the two mechanisms achieves superior overall antibacterial performance.

### 2.1 Mechanical bactericidal effects of micro/nanostructures

In both natural and artificial systems, surface micro/nanostructures play a crucial role in antibacterial performance by regulating bacterial behaviors. With advances in micro-/nano-fabrication technologies, increasing evidence has shown that the antibacterial activity of bioinspired and engineered surfaces is primarily governed by the mechanical sterilization effect of their micro/nanostructures.

#### 2.1.1 Bionic inspiration

The concept of mechanical bactericidal surfaces originates from observations of insect wings in nature. Dragonfly and cicada wings are covered with dense nanopillar arrays that can rapidly inactivate bacteria upon contact without the involvement of chemical agents (Hasan et al., 2013b; Li, 2016; Kelleher et al., 2016; Pogodin et al., 2013; Mainwaring et al., 2016; Ivanova et al., 2013). This antibacterial activity arises from purely physical interactions between bacterial cells and surface nanostructures, providing important inspiration for the development of bioinspired antibacterial materials.

**FIGURE 1:**
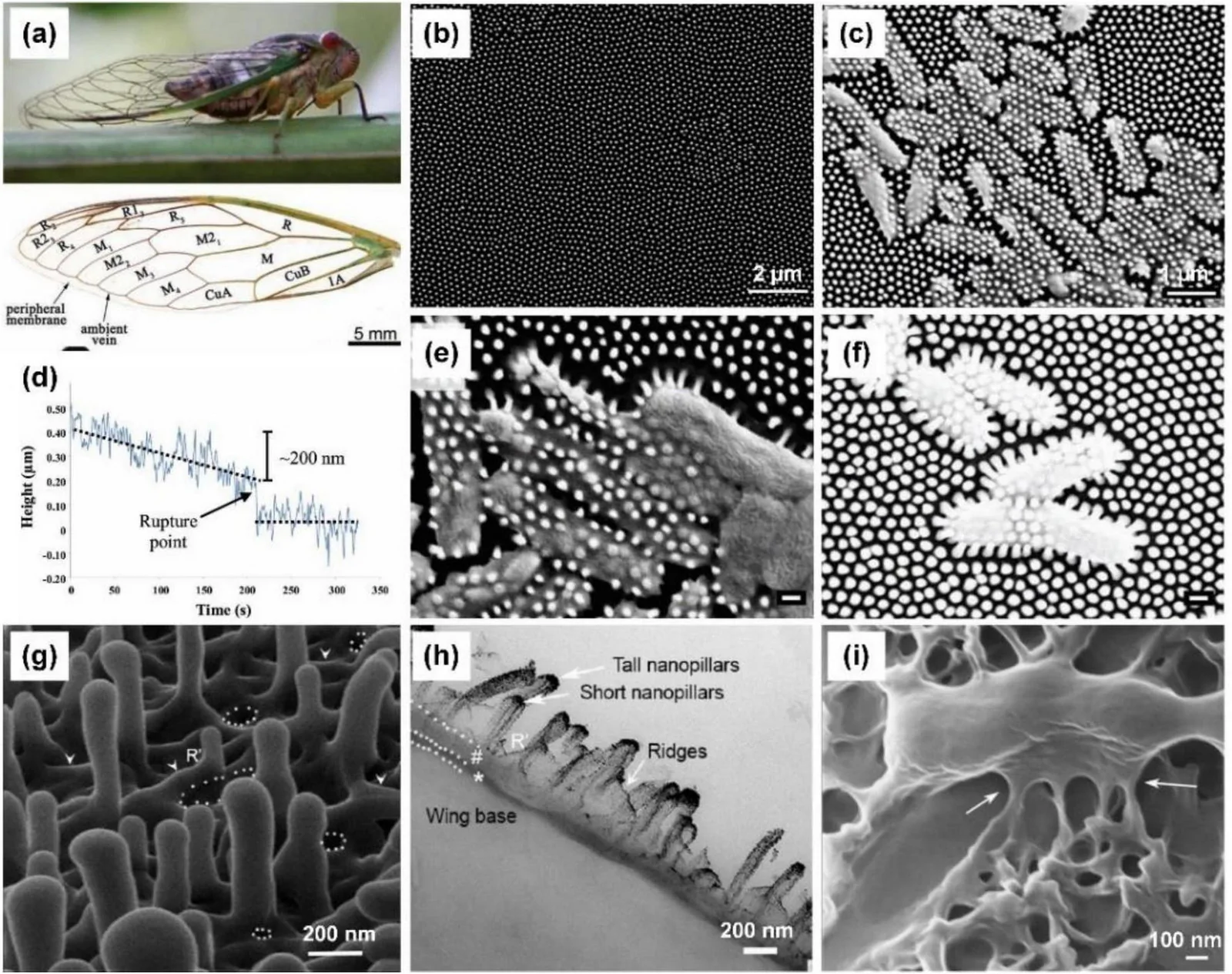
Nature mechanically bactericidal surfaces. **(a)** Photographs of a cicada and its forewing. **(b)** SEM image of the nanostructure array on the cicada wing surface. **(c)** *Pseudomonas aeruginosa* adhered to the cicada wing. **(d)** Time-resolved observation of bacterial cell rupture, indicating that membrane failure occurred approximately 3 min after contact. **(e,f)** SEM images of *Pseudomonas aeruginosa* on cicada wing nanostructures without (left) and with (right) a conformal gold coating. Reproduced with permission from Ivanova et al. (2012). Copyright 2012 Wiley. **(g,h)** Helium ion microscopy and transmission electron microscopy (TEM) images illustrating the nanocolumn arrangement on dragonfly wings. **(i)** Interaction between dragonfly wing nanocolumns and bacterial extracellular polymeric substances (EPS). Reproduced with permission from Bandara et al. (2017). Copyright 2017 American Chemistry Society.

Cicada (*Psaltoda claripennis*) wings were the first reported natural mechanically bactericidal surfaces (Figures 1a,b; Ivanova et al., 2012). Their nanopillar arrays can physically damage bacterial membranes, causing leakage of intracellular contents and cell death (Figures 1c,d). Studies using ultrathin gold coatings demonstrated that the antibacterial performance is independent of surface chemistry and is primarily governed by the surface nanostructure itself (Figures 1e,f).

Dragonfly (Orthetrum villosovittatum) wings exhibit a different nanostructural architecture and bactericidal mechanism (Figures 1g,h; Bandara et al., 2017). Rather than directly penetrating bacterial membranes, their nanostructures interact with extracellular polymeric substances (EPS) secreted by bacteria. The resulting adhesion and shear forces generate mechanical stress on the cell envelope, leading to membrane damage and bacterial death (Figure 1i). These findings highlight that natural nanostructures can achieve antibacterial activity through distinct physical pathways, including membrane puncture and stress-induced rupture.

#### 2.1.2 Mechanical bactericidal mechanisms

The discovery of bactericidal nanostructures on insect wings has stimulated extensive research into the mechanisms underlying bacteria–surface interactions. Current studies suggest that bacterial inactivation on nanostructured surfaces is primarily governed by two mechanisms: tensile rupture and puncture rupture. In the former, bacterial membranes are stretched between adjacent nanostructures and rupture when the induced strain exceeds the membrane’s mechanical limit (Linklater et al., 2018; Michalska et al., 2018; Ivanova et al., 2020; Zahir et al., 2020; Xue et al., 2015; Li and Chen, 2016). In the latter, sharp high-aspect-ratio nanostructures directly penetrate the bacterial envelope, causing membrane failure and cell death (Modaresifar et al., 2019; Ghosh et al., 2017; Wandiyanto et al., 2020; Modaresifar et al., 2020; Velic et al., 2021).

**FIGURE 2:**
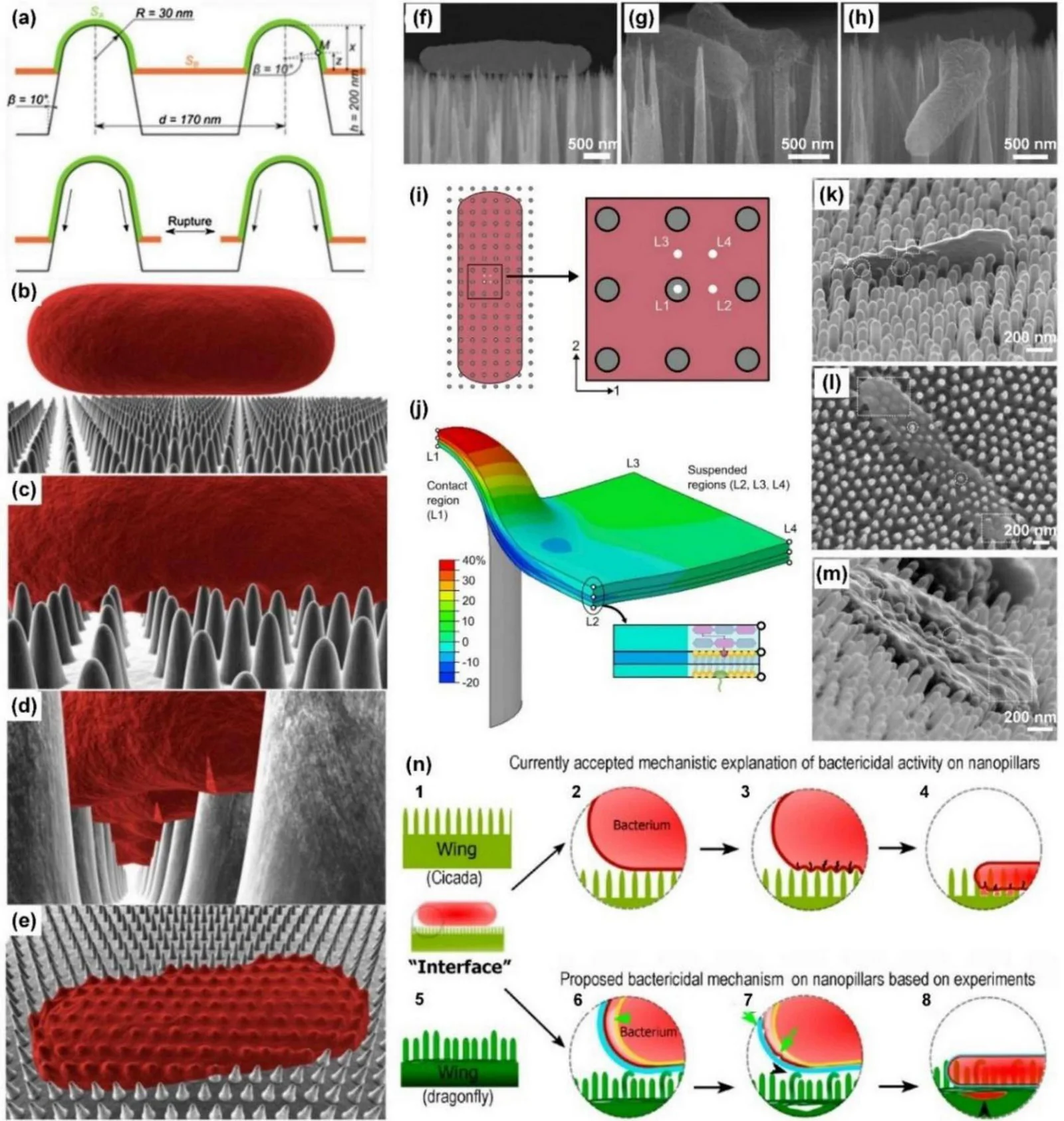
Fundamental principles of mechanically bactericidal surfaces. **(a)** Schematic illustration of the outermost layer of a bacterial cell adsorbed onto nanocolumns on a cicada wing surface. **(b–e)** Three-dimensional models illustrating the interaction between rod-shaped bacteria and the surface nanostructures of cicada wings. Reproduced with permission from Pogodin et al. (2013). Copyright 2013 Biophysical Society. **(f–h)** SEM images of bacteria adhered to black silicon surfaces featuring sharp nanocolumns. Reproduced with permission from Michalska et al. (2018). Copyright 2018 Royal Society of Chemistry. **(i)** Schematic diagram of the interaction between bacterial cells and surface nanocolumn arrays. **(j)** Uniaxial strain contour map depicting the interaction between the bacterial cell envelope and one-quarter of a nanocolumn structure. **(k)** Partially penetrated Gram-negative *Pseudomonas aeruginosa* cell retaining its overall morphology while exhibiting swelling, adhered to the nanopatterned surface of a cicada wing. **(l,m)** Fully swollen and penetrated bacterial cells adhered to the nanopattern on cicada wing surfaces. Reproduced with permission from Velic et al. (2021). Copyright 2021 Biophysical Society. **(n)** Schematic comparison of bactericidal mechanisms associated with nanocolumns on cicada and dragonfly wing surfaces: (1–4) Widely accepted models describing the bactericidal mechanism of nanocolumns on cicada wing surface; (5–8) bactericidal mechanism of nanocolumns on dragonfly wing surfaces. Reproduced with permission from Bandara et al. (2017). Copyright 2017 American Chemistry Society.

For the tensile rupture mechanism, bacterial adhesion to nanopillar arrays generates membrane deformation and tensile stress, particularly in regions suspended between neighboring nanostructures (Figures 2a–e; Pogodin et al., 2013). The resulting membrane stretching can eventually lead to rupture. Studies further indicate that structural parameters such as nanopillar height and spacing strongly influence the magnitude of membrane strain and bactericidal efficiency (Wu et al., 2018).

The puncture rupture mechanism proposes that sharp nanostructures concentrate stress at the bacteria–surface interface and physically pierce the cell envelope (Figures 2f–h; Michalska et al., 2018). Experimental observations and finite element simulations have shown that maximum strain is often localized at the nanopillar tips, supporting the possibility of direct membrane penetration and subsequent bacterial lysis (Figures 2i–m; Velic et al., 2021).

In addition to these two mechanisms, alternative pathways have also been proposed. For example, on dragonfly wings, bacterial cells interact with nanostructures through extracellular polymeric substances (EPS), generating adhesion- and shear-induced stresses that promote membrane delamination and rupture rather than direct penetration (Figure 2n; Bandara et al., 2017).

Overall, mechanical bactericidal surfaces provide a chemical-free antibacterial strategy with broad-spectrum activity, long-term stability, and a low risk of inducing antimicrobial resistance. These natural systems have inspired the development of bioinspired antibacterial interfaces, where structural parameters such as nanopillar height, spacing, and tip geometry can be optimized to enhance bactericidal performance for applications in medical devices, filtration systems, and high-contact surfaces.

### 2.2 Antibacterial effects of superhydrophobicity

Surface wettability plays a crucial role in regulating bacterial adhesion and biofilm formation (Hasan and Chatterjee, 2015; Yang et al., 2022; Zheng et al., 2021; Kreve and Dos Reis, 2022). By altering interactions at the bacteria–liquid–solid interface, surfaces can exhibit anti-adhesion, self-cleaning, and antibacterial functions (Martínez Gil-Ortega et al., 2025; Hsieh and Chien, 2023; Yin et al., 2016; Al-Amshawee et al., 2021). As a result, superhydrophobic surfaces have attracted significant attention in antibacterial materials research (Hasan et al., 2013a; Zhou et al., 2018; Zhang et al., 2013).

#### 2.2.1 Wettability theory

**FIGURE 3:**
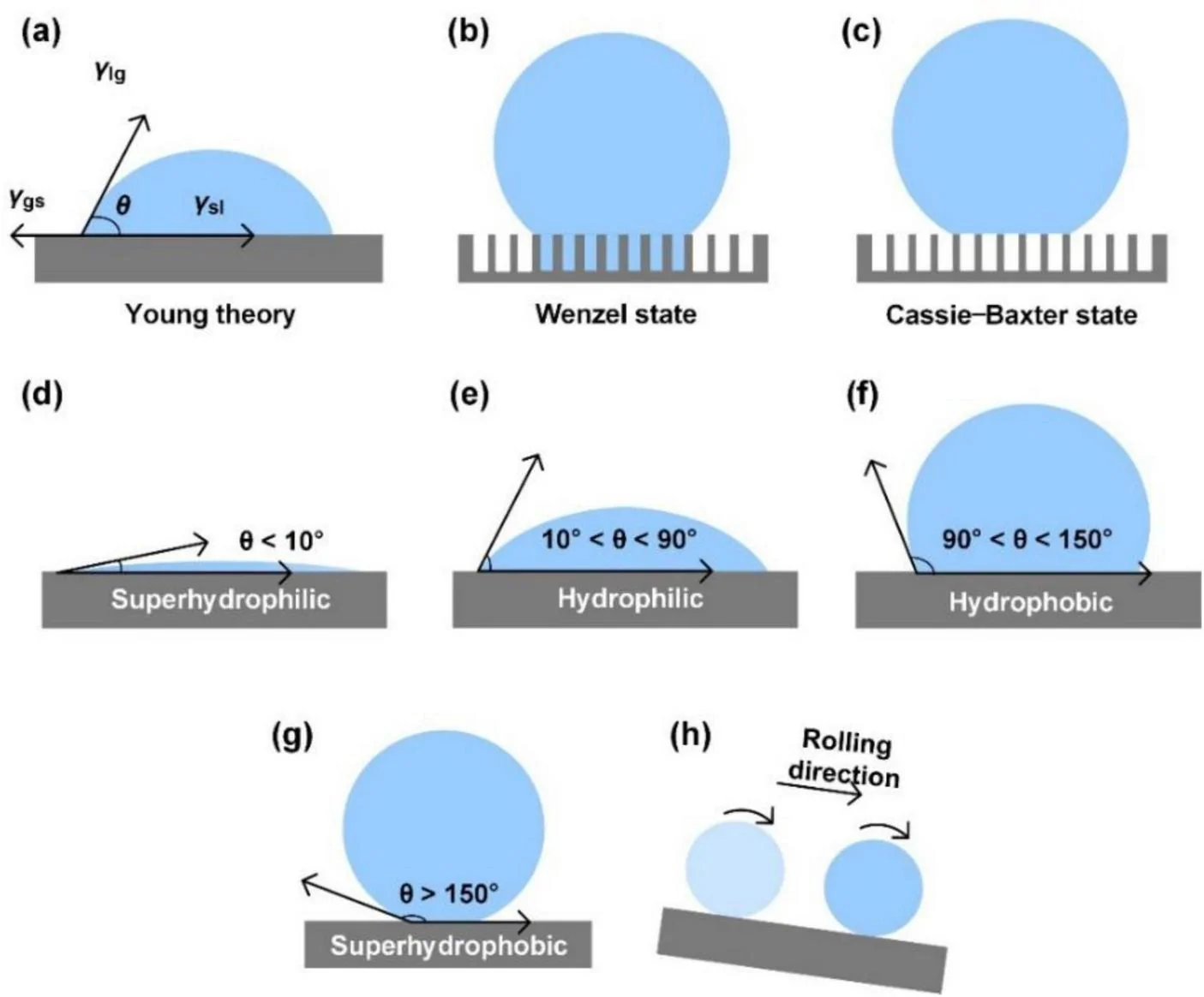
Theories related to wettability. **(a)** Young equation. **(b)** Wenzel state. **(c)** Cassie–Baxter state. **(d–g)** Definitions of contact angles on superhydrophilic, hydrophilic, hydrophobic, and superhydrophobic surfaces. **(h)** Superhydrophobic surface with a low rolling angle.

Wettability describes the interaction between a liquid and a solid surface and is commonly characterized by the water contact angle (Marmur et al., 2017). According to Young’s theory (Young, 1805), wettability quantitatively reflects the influence of a liquid on a solid surface by measuring the tangent angle at the solid–liquid–gas three-phase interface through the cross-sectional profile of a droplet (Figure 3a). Surfaces with contact angles below 90° are hydrophilic, whereas those above 90° are hydrophobic. Surfaces with contact angles greater than 150° are generally classified as superhydrophobic (Zhao and Jiang, 2018; Callies and Quéré, 2005).

In practical applications, surface roughness significantly affects wettability. The Wenzel model describes the wetting state in which a liquid completely penetrates surface microstructures, while the Cassie–Baxter model applies when air pockets are trapped within the surface texture, forming a composite solid–liquid–air interface (Figures 3b,c; Wenzel, 1936; Herminghaus, 2000; Cassie and Baxter, 1944). Compared with the Wenzel state, the Cassie–Baxter state greatly reduces the solid–liquid contact area and adhesion, resulting in enhanced water repellency. Figures 3d–g illustrates various wettability states of water droplets on solid surfaces, including superhydrophilic, hydrophilic, hydrophobic, and superhydrophobic.

Therefore, superhydrophobicity is typically achieved through the combination of low-surface-energy materials and hierarchical micro/nanostructures. In addition to a high contact angle, superhydrophobic surfaces generally exhibit a low sliding or rolling angle (Figure 3h), enabling efficient droplet mobility and self-cleaning behavior.

#### 2.2.2 Natural superhydrophobic surfaces and their antibacterial mechanisms

**FIGURE 4:**
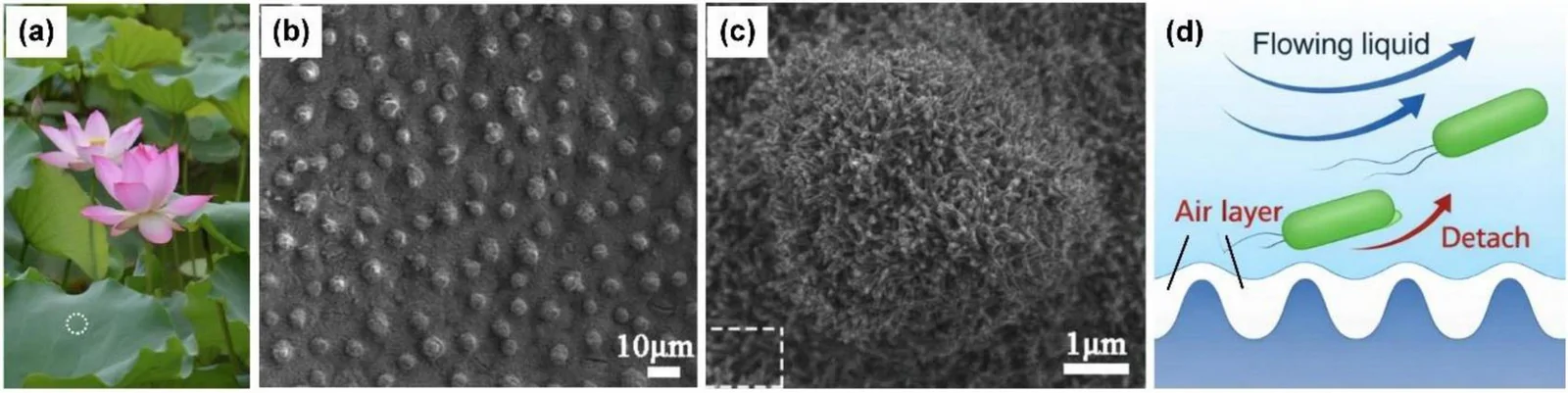
Special wettable surfaces in nature and their antibacterial principles. **(a–c)** Lotus leaves and SEM images of their surface structures. Reproduced with permission from Li et al. (2019). Copyright 2019 Elsevier B.V. **(d)** Schematic illustration of antibacterial mechanisms on superhydrophobic surfaces.

Many organisms have evolved superhydrophobic surfaces to adapt to complex environments. The lotus leaf is one of the most representative examples (Zorba et al., 2008; Feng et al., 2002; Wang and Jiang, 2007), exhibiting excellent water repellency and self-cleaning properties due to its hierarchical micro/nanostructure and low-surface-energy wax layer (Figures 4a–c; Guo et al., 2011; Li et al., 2019). Inspired by the lotus leaf effect, numerous natural superhydrophobic surfaces, such as rose petals (Feng et al., 2008), rice leaves (Cao et al., 2018), water strider legs (Hu et al., 2003; Gao and Jiang, 2004), moth eyes (Kuo et al., 2016; Dewan et al., 2012), and gecko feet (Xu et al., 2018; Liu et al., 2012).

Superhydrophobicity is generally achieved through the combination of micro/nanostructures and low-surface-energy materials, resulting in water contact angles above 150° and low rolling angles (Quéré, 2008; Wang et al., 2020; Khan et al., 2022). Under the Cassie–Baxter state, trapped air pockets minimize liquid–solid contact and create an effective barrier against bacterial adhesion. The antibacterial performance of superhydrophobic surfaces mainly arises from two mechanisms: (1) reduced bacterial attachment due to the presence of an air layer (Figure 4d; Truong et al., 2012), and (2) removal of contaminants and microorganisms through the self-cleaning effect of rolling water droplets (Zhan et al., 2022).

Notably, there exists a reasonable competitive coupling relationship between superhydrophobic anti-adhesion and mechanical bactericidal performance, rather than mutual inhibition. The air-layer-dominated Cassie state indeed reduces solid–bacteria contact, which weakens the mechanical piercing effect of sharp nanostructures. However, there are clear synergistic boundary conditions between the two functions. Under the fully stable Cassie state, the surface prioritizes anti-adhesion protection by blocking most bacterial invasion; although mechanical contact probability decreases, the ultra-low bacterial coverage effectively prevents biofilm coverage and structural passivation, thereby maintaining the long-term activity of mechanical bactericidal nanostructures. More importantly, the natural multiscale structure always maintains a partial wetting transition state between ideal Cassie and Wenzel modes: local air layer collapse enables discrete bacterial contact with structural peaks, while most regions still retain superhydrophobic antifouling characteristics. This specific boundary window achieves the optimal cooperative effect, which not only suppresses large-scale bacterial adhesion via air insulation, but also efficiently inactivates residual adherent bacteria through mechanical stress damage.

Owing to their chemical-free operation, self-cleaning capability, and long-term stability, natural-inspired superhydrophobic surfaces have shown great potential in biomedical and antifouling applications (Bartlet et al., 2018; Loo et al., 2012; Xun et al., 2020; Imani et al., 2019; Montgomerie and Popat, 2021; Magin et al., 2010; Rasitha et al., 2024; Tesler et al., 2015; Wang M. K. et al., 2021; Zhang et al., 2022). Their apparent long-term stability only holds under mild environments; performance degradation inevitably appears under harsh conditions such as high humidity, prolonged immersion, or mechanical wear, which mainly originates from continuous air plastron attenuation (Chen et al., 2025; Milionis et al., 2016). To tackle such intrinsic instability, emerging strategies including air-layer active regeneration and intrinsically stable aerophilic structural design have been proposed to extend service life, and these natural bionic structures still provide valuable design principles for the development of advanced long-lived antibacterial interfaces.

## 3 Synergistic design of micro/nanostructures and superhydrophobicity

Relying solely on a single microstructure design or surface chemical modification often fails to achieve both long-lasting anti-adhesion and highly efficient sterilization under prolonged usage conditions (Xu et al., 2021). Table 1 summarizes the advantages and disadvantages of various strategies, including the mechanical bactericidal effect of micro/nanostructures and superhydrophobic surfaces. In recent years, synergistic antibacterial strategies that combine micro/nanostructures with superhydrophobicity control have attracted increasing attention (Oopath et al., 2023). By regulating local stress through micro/nanostructures while using superhydrophobicity to control bacteria–interface interactions, this approach can achieve a dual synergy of mechanical sterilization and anti-adhesion, offering green and highly efficient antibacterial solutions for applications in medical devices, industrial equipment, and public health.

**TABLE 1:** Categories of single antibacterial strategies and their advantages and disadvantages.

Antibacterial strategy	Advantages	Disadvantages
Mechanical bactericidal effect	• Chemical-free antibacterial mechanism Low risk of inducing bacterial resistance • Broad-spectrum activity against Gram-positive and Gram-negative bacteria • Long-term durability due to structure-based functionality	• Strong dependence on micro/nanostructure geometry • Mechanical fragility of hierarchical structures • Potential damage to mammalian cells in biomedical applications • Accumulation of dead bacterial debris may reduce efficiency
Superhydrophobic surface	• Strong water repellency reduces bacterial adhesion • Self-cleaning effect removes contaminants and bacteria • Can reduce biofouling in aqueous environments • Anti-corrosion	• Loss of superhydrophobicity upon surface contamination • Limited effectiveness against bacteria in prolonged immersion • Mechanical fragility of hierarchical structures

### 3.1 Synergy principle

The synergistic antibacterial performance of micro/nanostructured superhydrophobic surfaces originates from the bidirectional coupling and mutually reinforcing interaction between hierarchical micro/nanoscale topography and superhydrophobic wetting state, rather than a simple linear superposition of independent anti-adhesion and bactericidal functions (Su et al., 2025; Senevirathne and Yarlagadda, 2025). On the one hand, the artificially constructed micro/nano hierarchical roughness serves as the fundamental structural prerequisite for stabilizing the superhydrophobic interface. The discrete microprotrusions and dense nanospines on the surface trap abundant air pockets within the texture, forming a robust solid–air–liquid composite interface. This unique wetting state drastically reduces the solid–bacteria contact area, suppresses initial bacterial anchorage and colonization, and endows the surface with passive antifouling and self-cleaning capability via rolling droplet removal. On the other hand, the residual individual bacteria that occasionally overcome the air-layer barrier and adhere to the structural peaks are mechanically damaged by the sharp nanostructures. Localized tensile, shear, and piercing stresses generated by rigid nanocolumns irreversibly rupture bacterial membranes, achieving efficient physical sterilization without chemical biocides and drug resistance risks.

More importantly, an intrinsic positive-feedback synergy loop exists between the two mechanisms. The superhydrophobic air layer effectively isolates massive bacterial coverage and biofilm encapsulation, preventing the buried failure and passivation of underlying micro/nanostructures and thereby maintaining long-term mechanical bactericidal activity. In return, the continuous mechanical elimination of residual adherent bacteria avoids biofilm-induced surface wettability degradation, stabilizing the durable superhydrophobic antifouling performance. This mutual promotion effect enables the integrated surface to simultaneously achieve prevention (superhydrophobic anti-adhesion) and eradication (microstructure mechanical sterilization). Such coordinated coupling behavior fundamentally breaks the performance limitation of single-function surfaces, where pure superhydrophobic surfaces suffer from inevitable residual bacterial adhesion while pure mechanical bactericidal structures are vulnerable to biofilm contamination and performance attenuation. Therefore, the micro/nanostructure–superhydrophobicity synergy produces a “1+1 > 2” comprehensive antibacterial advantage, realizing long-term, high-efficiency, and green antifouling and bactericidal effects.

### 3.2 Bio-inspired micro/nanostructured superhydrophobic surfaces

By designing high-aspect-ratio nanocolumns or nanospikes in combination with low-surface-energy coatings, a superhydrophobic state is achieved, reducing the contact points between bacteria and the substrate. Rolling droplets can then remove attached bacteria, providing a self-cleaning effect. The sharpness and spacing of the nanostructures directly influence puncturing efficiency and bactericidal performance, while superhydrophobicity ensures both self-cleaning and initial bacterial adhesion inhibition. Typical examples of such surfaces include damselfly wings, gecko skin, and lotus leaves.

**FIGURE 5:**
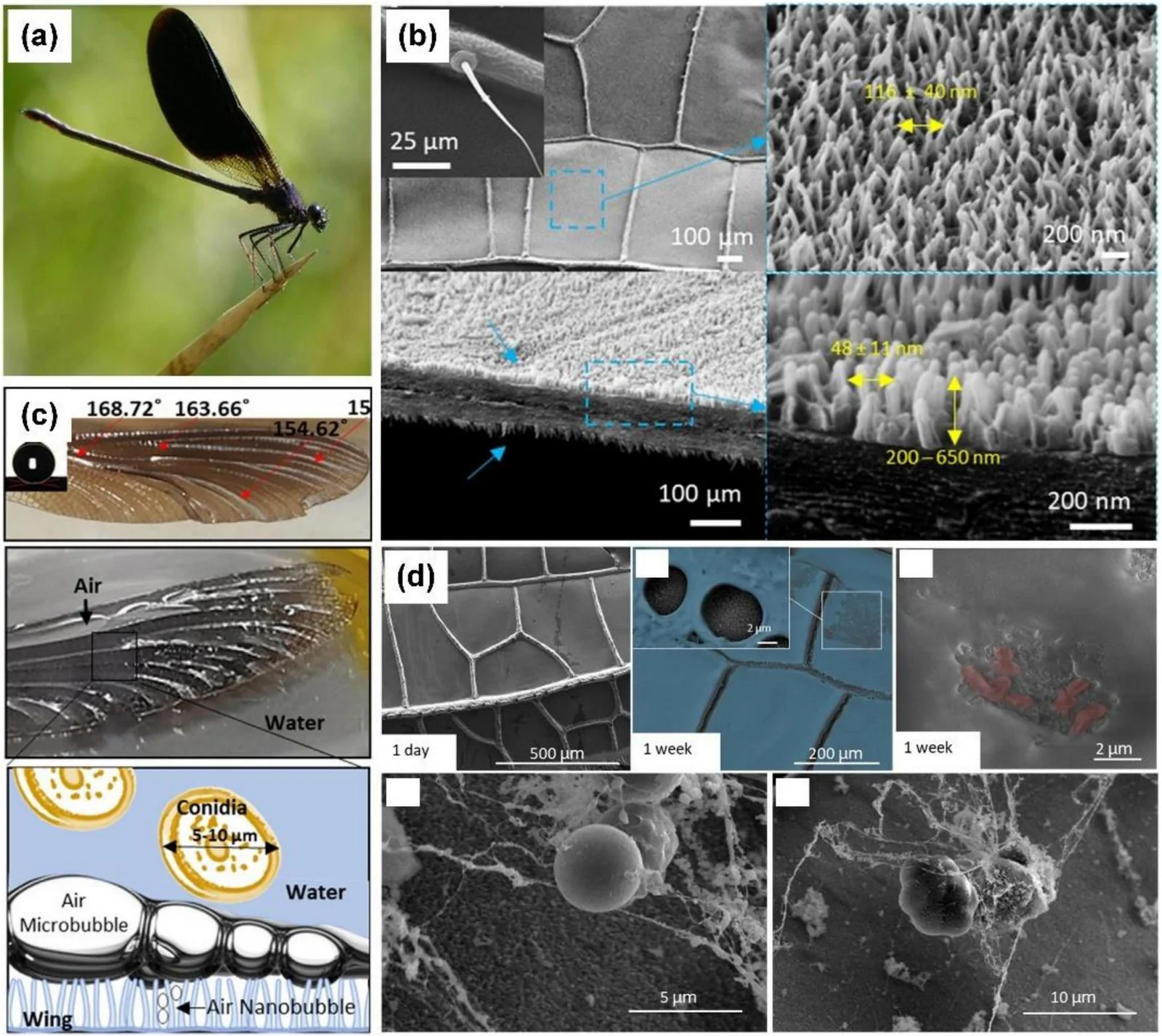
*Calopteryx haemorrhoidalis* wings and their antibacterial performance. **(a,b)** *Calopteryx haemorrhoidalis* and SEM images of the wing and its surface micro/nanostructures. **(c)** Superhydrophobic property of the wing, showing the air layer retained on the wing surface when immersed in water. **(d)** Cryo-scanning electron microscopy (Cryo-SEM) analysis of bacterial and fungal interactions on the surface of the wing. Reproduced with permission from Ivanova et al. (2021). Copyright 2021 Elsevier Inc.

*Calopteryx haemorrhoidalis*, a close relative of dragonflies, possesses a disordered array of nanocolumns on its wing surface, with an average height of 433 ± 71 nm, diameter of 48 ± 11 nm, and spacing of 116 ± 40 nm (Figures 5a,b; Ivanova et al., 2021). The irregular distribution increases surface complexity, hindering stable microbial attachment. In combination with the epidermal wax layer, these nanostructures confer superhydrophobicity (water contact angle ≈ 155°–170°, Figure 5c), further reducing microbial adhesion. The wings exhibit distinct antimicrobial mechanisms against different microorganisms: bacterial cells are mechanically disrupted by the nanocolumns, whereas fungal spores are prevented from contacting the surface by the trapped air layer. Notably, fungal attachment remains inhibited even after 1 week of water immersion (Figure 5d). These characteristics offer valuable insights for the development of durable bioinspired antifouling and antibacterial surfaces.

**FIGURE 6:**
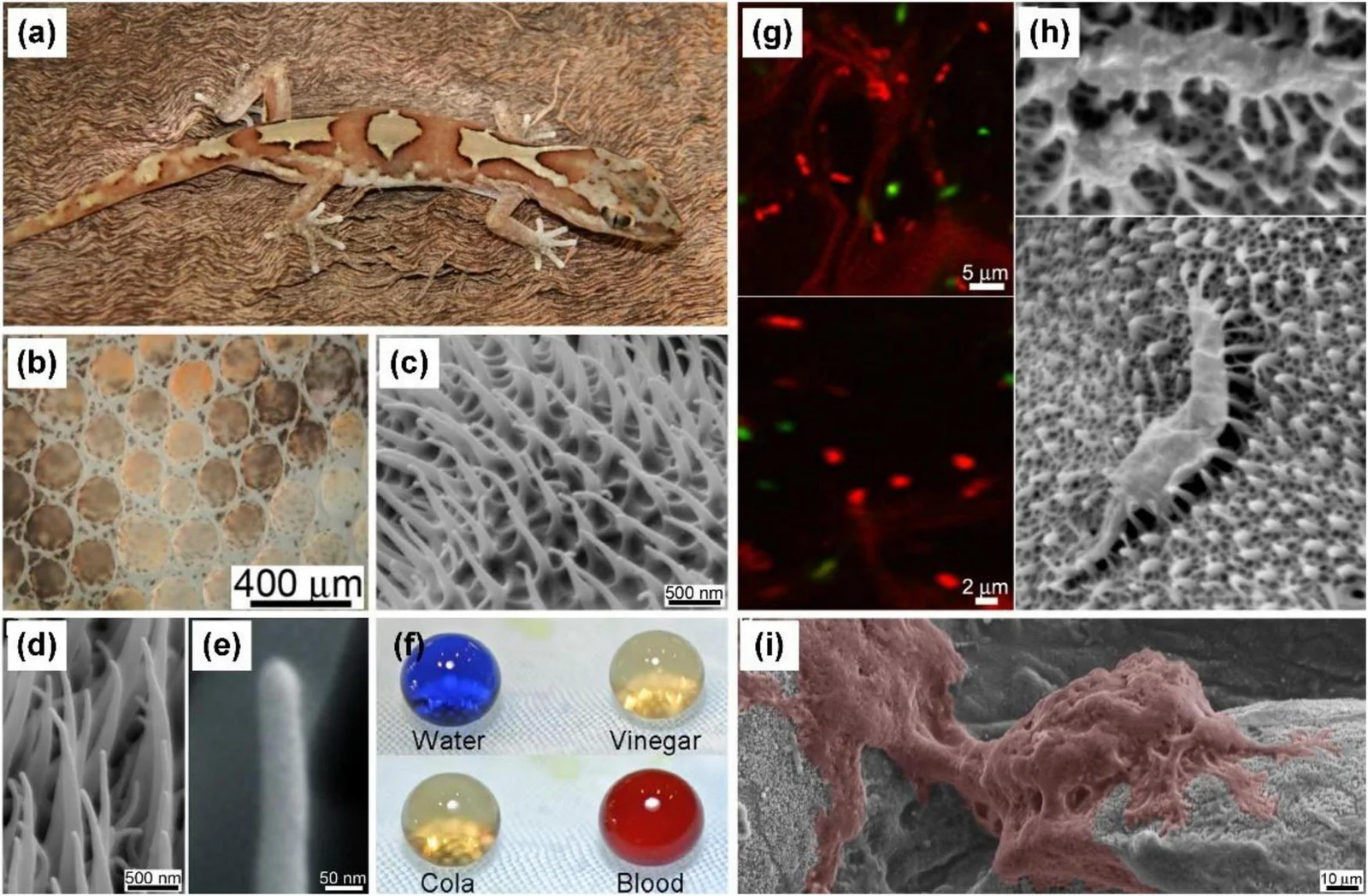
Gecko skin and its antibacterial performance. **(a–e)** Gecko and SEM images of the micro/nanostructures on the surface of gecko skin. **(f)** Superhydrophobic property of the gecko skin surface. **(g)** Confocal laser scanning microscopy (CLSM) images of *Porphyromonas gingivalis* on the gecko skin, with green indicating live cells and red indicating dead cells. **(h)** SEM images of the interaction between bacteria and the nanospike tips. **(i)** Dental pulp stem cells cultured on the gecko skin surface, with staining highlighting cell edges. Reproduced with permission from Watson et al. (2015). Copyright 2015 Elsevier Ltd.

Watson et al. (2015) investigated the hierarchical micro/nanostructure of gecko skin, which consists of hexagonally arranged dome-shaped scales covered with nanoscale spines (Figures 6a–e). This structure imparts excellent superhydrophobicity (Figure 6f), preventing water-film formation and reducing microbial adhesion while promoting self-cleaning. In addition, gecko skin exhibits significant antibacterial activity against *Porphyromonas gingivalis*, as the nanospines mechanically stretch and rupture bacterial cell walls (Figures 6g–i). The synergistic effects of micro/nanostructures and superhydrophobicity enable both self-cleaning and antibacterial functions, offering valuable inspiration for the development of biomimetic multifunctional surfaces and antibacterial materials.

**FIGURE 7:**
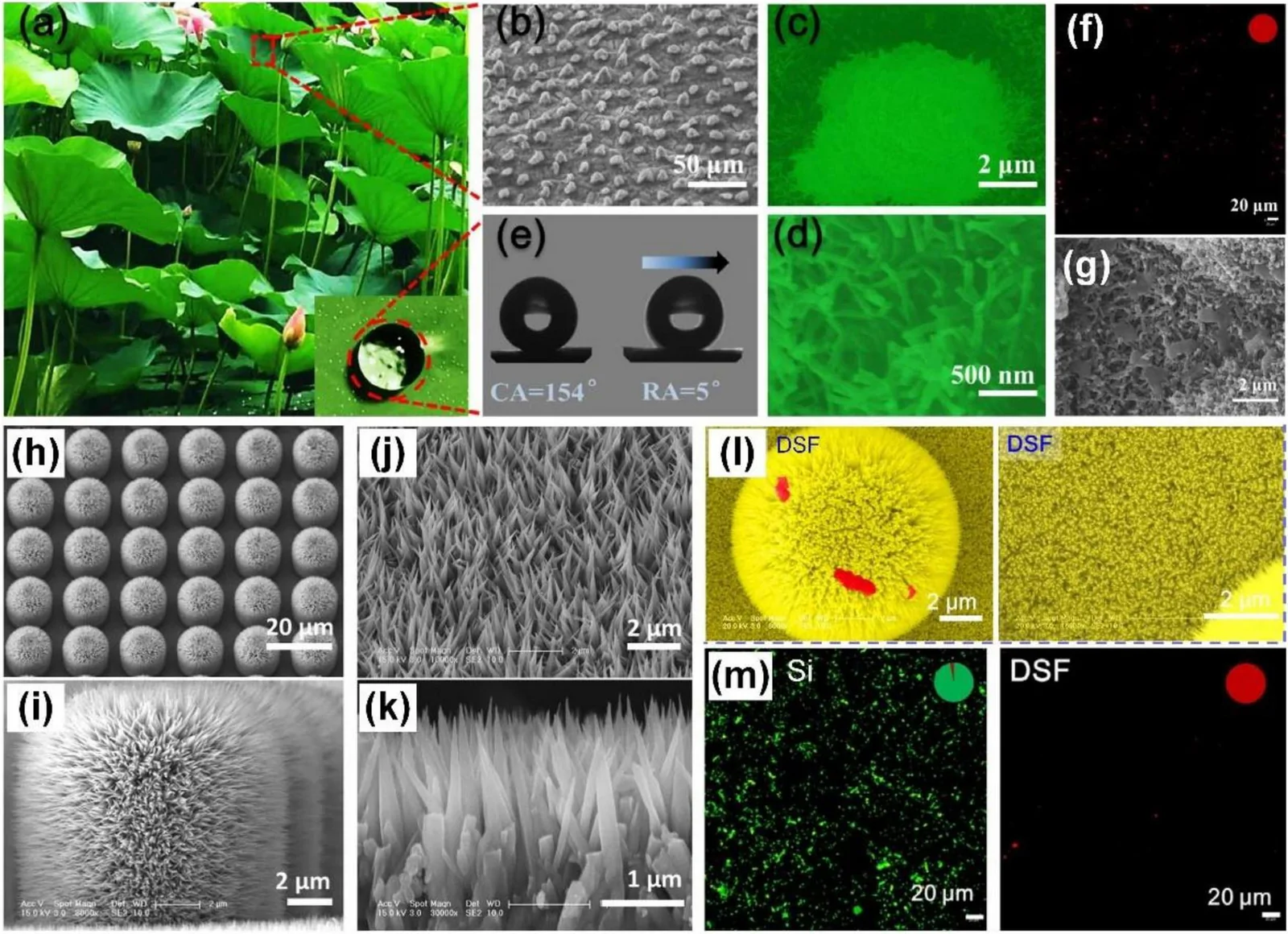
Antibacterial performance of natural lotus leaf surfaces and lotus leaf-inspired surfaces. **(a–d)** SEM images of lotus leaves and their surface micro/nanostructures. **(e)** Demonstration of the superhydrophobic property of the lotus leaf surface. **(f,g)** SEM and CLSM images of *Escherichia coli* after incubation on the surface of fresh lotus leaves for 24 h. **(h–k)** SEM images of the lotus leaf-inspired micro/nanostructures fabricated on the artificial surface. **(l)** SEM images showing the morphology of *Escherichia coli* cells adhering to the sample after 24 h of incubation. DSF denotes the modified micro/nanostructured surface mimicking lotus leaves. **(m)** CLSM images of *Escherichia coli* after 24 h of incubation on a silicon surface (control sample) and on the DSF surface. Reproduced with permission from Jiang et al. (2020). Copyright 2020 Elsevier B.V.

Jiang et al. (2020) investigated the antibacterial mechanism of lotus leaves and revealed the synergistic effects of hierarchical micro/nanostructures and superhydrophobicity. The lotus leaf surface consists of micrometer-scale papillae covered with high-aspect-ratio nanotubules (Figures 7a–d), resulting in a high water contact angle and low rolling angle (Figure 7e). After exposure to *Escherichia coli* for 3 and 24 h, nearly all adhered bacteria were inactivated (Figures 7f,g). This antibacterial performance arises from the combined action of self-cleaning and mechanical sterilization: rolling droplets remove most bacteria, while the nanotubular structures physically damage cells that directly contact the surface. Inspired by this mechanism, a biomimetic hierarchical surface was fabricated and fluorinated to achieve excellent superhydrophobicity (Figures 7h–k). The resulting surface effectively repelled most bacteria and mechanically killed the few that adhered (Figures 7l,m), demonstrating durable antibacterial performance without the use of chemical biocides.

**FIGURE 8:**
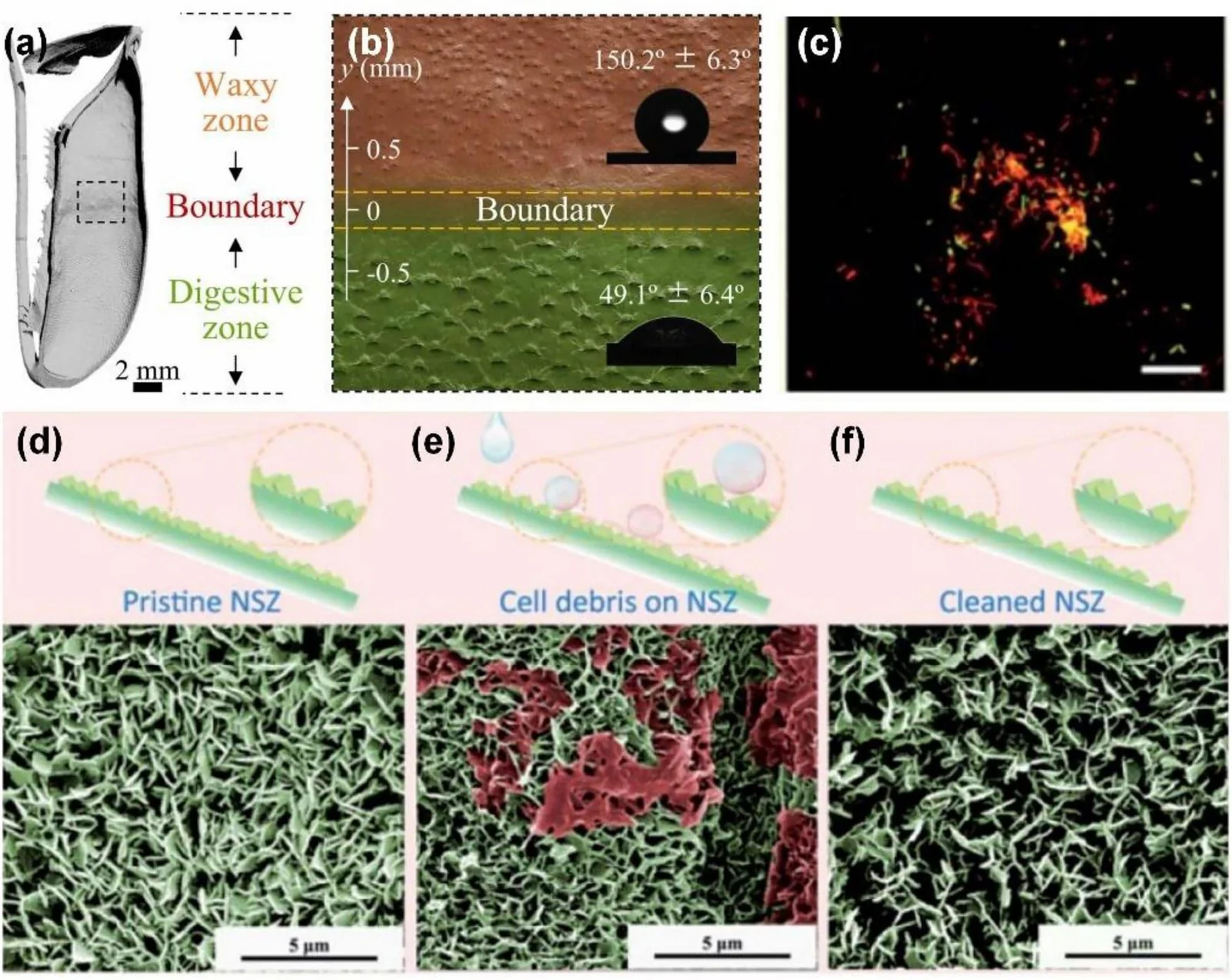
Antibacterial properties of the superhydrophobic waxy zone of *Nepenthes*. **(a,b)** *Nepenthes* and the superhydrophobic characteristics of its waxy regions. Reproduced from Ma et al. (2025). Copyright 2025 The Authors, published by AAAS under a CC BY-NC license. **(c)** CLSM image of *Escherichia coli* attached to the waxy region of *Nepenthes*, showing effective bacterial inactivation by the waxy-zone nanostructures. **(d)** SEM image of the nanostructures in the waxy region. **(e)** Residual debris of inactivated *Escherichia coli* remaining on the surface of the waxy-zone nanostructures. **(f)** Self-cleaning behavior of the nanostructured waxy surface after contamination, where the *E. coli* debris has been completely removed. Reproduced from Xie et al. (2019) with permission from the Royal Society of Chemistry. Copyright 2019.

Superhydrophobic self-cleaning surfaces not only remove adhered bacteria but also eliminate dead-cell debris, re-exposing bactericidal nanostructures and sustaining antibacterial activity. The waxy region on the inner wall of pitcher plants exhibits pronounced superhydrophobicity (Figures 8a,b; Ma et al., 2025). Xie et al. (2019) found that this surface efficiently inactivates *Escherichia coli* through densely distributed leaf-like wax crystals (Figures 8c–e), whose sharp edges physically disrupt bacterial membranes. Meanwhile, the superhydrophobic self-cleaning effect rapidly removes bacterial debris, maintaining continuous bactericidal performance (Figure 8f). Inspired by this mechanism, researchers developed a multifunctional surface based on Zn–Al layered double hydroxide nanoblades, combining mechanical bactericidal activity with self-cleaning capability. After fluorosilane modification, the surface showed efficient antibacterial performance against both *E. coli* and *Staphylococcus aureus* with minimal debris accumulation after repeated sterilization cycles, demonstrating excellent durability and reusability.

In conclusion, the synergistic integration of micro/nanostructures and superhydrophobicity delivers a bioinspired interfacial regulation strategy derived from lotus leaves, damselfly wings and gecko skin. Such surfaces reduce bacterial attachment by weakening bacteria-substrate contact and physically kill adherent bacteria via sharp nanostructures, integrating superhydrophobic self-cleaning and mechanical bactericidal effects to realize chemical-free, eco-friendly bacterial inhibition. Table 2 summarizes the sterilization efficiency of different structural types (including natural and artificial antibacterial surfaces). Nevertheless, conventional discussions overly focus on direct bacterial elimination, while overlooking realistic natural environmental conditions. In practical service scenarios, surfaces are constantly bombarded by continuous microbial colonization, organic conditioning films and cellular metabolic wastes. These contaminants gradually cover delicate nanostructures, degrade superhydrophobic properties, and ultimately trigger biofilm regeneration. Therefore, these surfaces can only alleviate and slow down surface fouling instead of offering permanent anti-fouling protection. More accurately, this technology belongs to fouling management rather than absolute fouling prevention. Given the inherent limitations of passive superhydrophobic interfaces, the development trend of next-generation functional surfaces is shifting toward active stimuli-responsive designs. Such smart surfaces can dynamically and long-term modulate biofilm proliferation, which will lay a new theoretical framework for the long-term stable application of advanced biomimetic antibacterial interfaces.

**TABLE 2:** Sterilization efficiency of different structural types including natural and artificial antibacterial surfaces.

Material	Structure (type)	Structure (dimensions)	Wettability	Bacterial strain	Antibacterial efficiency	Reference
Cicada wing	Hexagonally packed conical nanopillars with spherical caps	Nanopillar height: 200 nm Base diameter: 100 nm Spherical cap diameter: 60 nm Pitch: 170 nm	Contact angle: 158.8°	*Pseudomonas aeruginosa*	Single bacterial cell killed within ∼3 min of contact	Ivanova et al., 2012
Dragonfly wing	Cylindrical nanopillars	Short nanopillars: Height 189 ± 67 nm, diameter 37 ± 6 nm Long nanopillars: Height 311 ± 52 nm, diameter 57 ± 8 nm	Hydrophobic (degree not specified)	*Escherichia coli*	1.65 × 10^5^ cells min^–1^ cm^–2^	Bandara et al., 2017
Damselfly wing	Nanopillar array	Average nanopillar height: 433 ± 71 nm Nanopillar diameter: 48 ± 11 nm Pillar spacing: 116 ± 40 nm	Contact angle: 155°–170°	*Pseudomonas aeruginosa*, *Aspergillus brasiliensis*, *Epicoccum nigrum*	Not quantified (qualitative)	Ivanova et al., 2021
Gecko skin	Dome-shaped scales with spinules	Dorsal scale diameter: 100–190 μm, pitch similar, height > 50 μm Ventral scale diameter: up to 300 μm Spinule length: 4 μm	Contact angle: 151°–155°	*Porphyromonas gingivalis*	Not quantified (qualitative)	Watson et al., 2015
Lotus leaf and silicon wafer	Lotus leaf: Micro-papillae with nanotubules Silicon wafer: Microcylinder array with ZnO nanoneedles	Lotus leaf: Papilla diameter: ∼5–10 μm; Wax tubule length: ∼0.3–1.1 μm, diameter: ∼0.1–0.2 μm Silicon wafer: Microcylinder: Diameter ∼5 μm, height ∼13 μm, pitch ∼12 μm; ZnO nanoneedles: Tip diameter 30–50 nm, average height ∼2–2.5 μm	Lotus leaf: Contact angle = 154 ± 5°, roll-off angle = 5 ± 2° Silicon wafer: Contact angle = 174.0°, roll-off angle < 1°	*Escherichia coli*	Lotus leaf: Near-zero attachment at 3 h; near-total death of attached bacteria within 24 h Si wafer: Bactericidal efficiency > 98%	Jiang et al., 2020
Aluminum alloy (5A06)	Nanoblades	Blade tip width: 29.3 ± 5.7 nm Density: 4.4 ± 0.2 × 10^9^ blades cm^–2^	Contact angle: 148.6° and 144.3°	*Escherichia coli, Staphylococcus aureus*	E. coli: 2.10 ± 0.36 × 10^4^ CFU cm^–2^ min^–1^ S. aureus: 1.78 ± 0.47 × 10^3^ CFU cm^–2^ min^–1^	Xie et al., 2019

## 4 Applications of antibacterial surfaces

**FIGURE 9:**
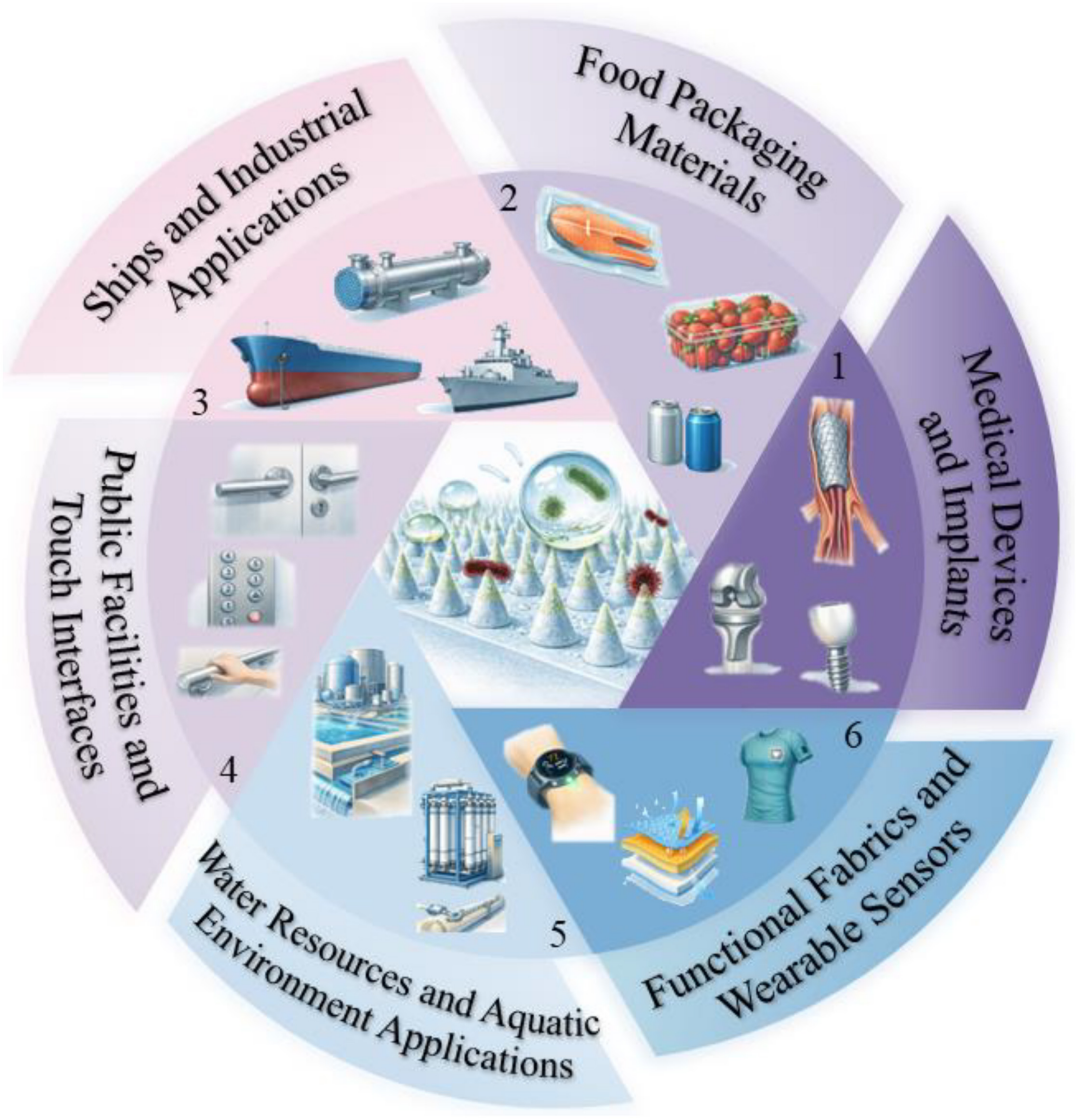
Potential applications of micro/nanostructure–superhydrophobic synergistic antibacterial surfaces.

Micro/nanostructure–superhydrophobic synergistic anti-bacterial technology has demonstrated great potential across a wide range of application fields due to its advantages, including the absence of chemical agents, broad-spectrum antibacterial activity, and high durability. Typical applications span medical devices, food packaging, industrial anti-fouling, public facilities, water resources and aquatic environments, functional textiles, and wearable sensors (Figure 9).

### 4.1 Medical devices and implants

Medical devices and implants, such as catheters, cardiovascular stents, artificial joints, and orthopedic implants, are highly susceptible to bacterial adhesion and biofilm formation during surgical procedures and long-term implantation, significantly increasing the risk of postoperative infections (Zhang et al., 2021; Wang et al., 2024; Yu et al., 2024; Al-Suhaimi et al., 2026). Micro/nanostructure–superhydrophobic synergistic antibacterial strategies play a crucial role in mitigating these risks when applied to the surfaces of medical devices. Mechanical bactericidal effects induced by micro/nanostructures, such as sharp nanocolumns or nanospikes, can physically disrupt bacterial cell membranes upon contact, enabling efficient sterilization without reliance on antibiotics or chemical agents (Zacher et al., 2024). In addition, superhydrophobic surfaces can form stable trapped air layers, respectively, which effectively reduce bacterial adhesion and enhance surface self-cleaning performance (Liu et al., 2025). However, most indwelling medical devices are permanently immersed in physiological fluids. Long-term hydrostatic pressure, continuous fluid scouring, and gradual gas diffusion will collapse the trapped air layer, triggering an irreversible transition from the Cassie-Baxter state to the Wenzel state. This wettability failure completely eliminates the anti-adhesion effect, greatly weakening the *in vivo* antibacterial and anti-biofilm performance of the micro/nano superhydrophobic coating.

Nevertheless, two key challenges hinder clinical translation. First, the delicate hierarchical micro/nanostructures are mechanically fragile; *in vivo* friction, tissue extrusion, and blood scouring easily cause structural damage and peeling, leading to functional failure and poor long-term implant durability. Future work should develop gradient and interface-reinforced structural designs to balance antibacterial activity and mechanical stability. Second, rigid bactericidal micro/nanostructures may induce mechanical damage to normal human cells and surrounding tissues, triggering inflammation and biocompatibility risks. Further research is required to optimize structural morphology and dimensional parameters to construct bacteria-specific inactivation interfaces, eliminating cytotoxicity while maintaining high antibacterial efficiency for clinical safety.

### 4.2 Food packaging materials

Microbial contamination on the surfaces of food packaging materials directly affects food safety and shelf life (Siroli et al., 2017; Pandey et al., 2022). Conventional chemical antibacterial packaging often suffers from issues such as uneven release of antimicrobial agents, potential development of microbial resistance, and environmental pollution (Shoba et al., 2025; Upadhyay et al., 2024). In contrast, micro/nanostructure–superhydrophobic synergistic antibacterial strategies provide a green and sustainable alternative. Superhydrophobic packaging surfaces allow rolling water droplets to remove adhered bacteria, thereby reducing microbial accumulation on food-contact surfaces (Li Z. L. et al., 2025; Liu et al., 2021; Oh et al., 2019; Yin et al., 2025; He R. et al., 2025; Yang et al., 2023). Additionally, engineered micro-nano topographies decrease the available attachment sites for bacteria, effectively inhibiting their adhesion, proliferation, and growth within packaging systems (Tian et al., 2025; Rajab et al., 2019).

Current research lacks systematic verification under practical food processing and storage conditions. Complex environments involving oil contamination, organic residues, repeated friction, and high humidity can damage micro/nanostructures and destroy superhydrophobic air layers, causing rapid functional failure. Moreover, organic and microbial metabolites readily accumulate within structural gaps, forming persistent hidden contamination that cannot be eliminated by conventional self-cleaning. Future studies should develop wear-resistant, anti-fouling composite micro/nanostructures and optimize surface energy and pore sizes to sustain stable superhydrophobicity and long-term surface cleanliness for food packaging applications.

### 4.3 Ships and industrial applications

Marine structures and industrial equipment, such as ship hulls, heat exchangers, and pipelines, are frequently plagued by the adhesion of algae, shellfish, and bacterial biofilms (Cao and Cao, 2023; Wu et al., 2025; Xia M. Q. et al., 2025; Deumic et al., 2025; Wang et al., 2026). This biofouling not only increases hydrodynamic resistance and energy consumption but also accelerates material corrosion and equipment degradation. Although traditional chemical anti-fouling coatings can be effective, they often suffer from drawbacks including toxicity and environmental pollution (Qiao et al., 2024). In this context, micro/nanostructure–superhydrophobic synergistic antibacterial technologies offer a sustainable and environmentally friendly alternative for industrial fouling control. Superhydrophobic micro/nanostructures can trap air layers on surfaces, effectively inhibiting the attachment of microorganisms, while hydrodynamic scouring further facilitates surface self-cleaning (Zhang et al., 2025; Yusuf et al., 2023; Selim et al., 2020). As a result, such surfaces can keep industrial equipment in a low-fouling state over extended periods, significantly enhancing service life and maintenance efficiency. Nevertheless, typical marine and pipeline equipment operates under permanent full immersion. Sustained hydrostatic pressure, flowing water shear force, and spontaneous gas diffusion gradually dissipate the interfacial air layer, switching the surface from the Cassie-Baxter state to the sticky Wenzel state. Such transition fundamentally abolishes the superhydrophobic anti-fouling advantage, making the coatings ineffective for long-term submerged anti-biofouling applications.

Combined with wettability state failure, poor environmental adaptability and structural durability limit large-scale industrial deployment. Harsh working conditions including long-term seawater immersion, high-speed fluid scouring, and dust erosion easily induce abrasion, structural collapse, and peeling of micro/nanostructures, attenuating superhydrophobic and anti-fouling performance. In addition, inorganic sludge and marine sediments block structural gaps and destroy interfacial air layers, causing irreversible fouling. Future research should focus on high-stability composite structural modification and self-repairing design to achieve durable anti-fouling and self-cleaning functions under extreme industrial and marine environments.

### 4.4 Public facilities and touch interfaces

Public facilities such as elevator buttons, door handles, handrails, and touch screens are frequently contacted by users and are therefore prone to acting as transmission media for pathogenic microorganisms (Reynolds et al., 2005; Appiah et al., 2025; Di Battista, 2022; Ackerley et al., 2019; Bhatta et al., 2018). Through the synergistic design of micro/nanostructures and surface wettability, the bacterial load on these high-touch interfaces can be effectively reduced, while rolling water droplets remove residual microorganisms via a self-cleaning effect (Agbe et al., 2020; Birkett et al., 2022; Chen et al., 2023b). These self-cleaning and low-maintenance characteristics further decrease cleaning frequency, enhance durability, and provide reliable protection for public health.

However, existing studies focus only on short-term antibacterial effects, ignoring long-term durability in high-usage scenarios. Frequent human friction, dust deposition, and daily detergent erosion damage delicate micro/nanostructures and degrade superhydrophobicity, leading to gradual functional failure. Accumulated sweat, dust, and microbial residues also form stubborn surface fouling. Future work should optimize structural wear resistance and stain resistance to maintain stable antibacterial and self-cleaning performance for long-term public health protection.

### 4.5 Water resources and aquatic environment applications

In water treatment systems, water distribution networks, and membrane separation equipment, the formation of bacterial and microbial biofilms is a major contributor to flux decline, water quality degradation, and equipment failure (Nguyen et al., 2012; Dreszer et al., 2014; Zhou et al., 2025). Conventional chemical sterilization and cleaning strategies typically rely on strong oxidants or antimicrobial agents, leading to increased operational costs and potential risks of secondary pollution (Slattery and Garvey, 2025; Wang Z. K. et al., 2021; Ke et al., 2024). Micro/nanostructure–superhydrophobic synergistic antibacterial surfaces offer a predominantly physical and environmentally friendly alternative for water resource and aquatic environment applications. By constructing micro/nanostructures on membrane materials or the inner walls of pipelines, the number of initial microbial attachment sites can be effectively reduced, thereby inhibiting biofilm nucleation and growth (Kim et al., 2022). The application of such surfaces in oil–water separation and water transportation systems is expected to substantially mitigate membrane fouling, enhance water treatment efficiency, and improve long-term operational stability (Wang et al., 2022; Ghadei et al., 2025; Wang et al., 2025; Dai and Fan, 2024; Liu et al., 2024; Li X. et al., 2025).

However, core water treatment facilities including filtration membranes and underground pipelines work in permanent full-submersion environments. Persistent hydrostatic pressure and continuous water flow destroy the fragile trapped air layer, leading to spontaneous conversion from Cassie-Baxter superhydrophobicity to the Wenzel wetting state. The loss of air-layer-based anti-adhesion capability severely compromises the long-term anti-biofouling stability of such surfaces in aquatic operational systems. Moreover, complex aquatic environments pose major challenges to structural stability and anti-fouling sustainability. Suspended particles, organic colloids, and microbial metabolites easily adsorb onto micro/nano surfaces, block structural gaps, and destroy superhydrophobic air layers. Long-term water scouring and microbial erosion also gradually damage hierarchical architectures and degrade equipment operational stability. Future research should develop anti-clogging, erosion-resistant micro/nano structures with enhanced substrate binding force to sustain long-term surface cleanliness and improve the service life of water treatment systems.

### 4.6 Functional fabrics and wearable sensors

Fabrics and functional textiles are prone to contamination by sweat, sebum, and microorganisms during prolonged wear or use, which can result in unpleasant odors, cross-infection, and degradation of material performance (Møllebjerg et al., 2021; Callewaert et al., 2014). Conventional antibacterial textiles predominantly rely on chemical antimicrobial agents, which often suffer from limited durability and potential health concerns (Morais et al., 2016; Karypidis et al., 2023; Novi et al., 2022). In contrast, micro/nanostructure–superhydrophobic synergistic antibacterial strategies provide a physical and sustainable approach for fabric surface functionalization (Suryaprabha et al., 2023; Kwon et al., 2020; Dalawai et al., 2020). By introducing micro/nanostructures onto fiber or fabric surfaces, the probability of bacterial adhesion at fiber junctions can be significantly reduced, thereby disrupting microbial colonization. When combined with superhydrophobicity, textile surfaces can achieve rapid sweat transport and self-cleaning behavior, allowing water and contaminants to detach efficiently under gravity or body motion. Moreover, bioinspired superhydrophobic structures help minimize microbial residue while preserving fabric softness and breathability, enabling long-lasting and reliable antibacterial performance in sportswear, medical protective textiles, and smart fabrics (Meng et al., 2025).

Wearable sensors and flexible electronic devices typically require prolonged and intimate contact with the skin, making them susceptible to sweat accumulation, skin microbiota, and external contaminants (Kim et al., 2019; Li et al., 2022; Cui et al., 2022). These factors can lead to signal drift, performance degradation, and potential biosafety risks. Micro/nanostructure–superhydrophobic synergistic antibacterial surfaces offer distinct advantages in this context. The incorporation of micro/nanostructures onto sensor encapsulation layers or flexible substrates effectively suppresses bacterial adhesion without compromising device flexibility or signal transmission (Yang J. X. et al., 2025; Huang et al., 2025). Such synergistic surface engineering not only enhances the antibacterial and anti-fouling properties of wearable devices but also ensures their reliability in long-term health monitoring and continuous-wear applications.

Two core bottlenecks restrict practical wearable applications: structural durability and skin biosafety. First, rigid micro/nano architectures are prone to cracking and peeling under repeated bending and stretching of flexible substrates, resulting in lost superhydrophobic and antibacterial functions. Future work requires flexible, deformable-compatible structural designs to enhance fatigue resistance. Second, improperly structured surfaces may cause mechanical irritation and damage to skin cells during long-term contact. Subsequent research should optimize bionic flexible structures to balance high antibacterial performance and skin biocompatibility for safe, long-term wearable usage.

In summary, micro/nanostructure–superhydrophobic synergistic antibacterial technologies exhibit versatile application prospects across medical, food, industrial, public facility, water environment, and wearable fields. Current laboratory research cannot fully address practical challenges including structural fragility, persistent surface fouling, and scenario-specific biosafety risks. Future studies should prioritize multi-scale structural synergy, long-term superhydrophobic stability, multifunctional integration, and field performance validation. Solving these key bottlenecks will advance the large-scale engineering implementation of such eco-friendly, high-stability functional interfacial materials.

## 5 Challenges and prospects

This review systematically summarizes recent advances in micro/nanostructure–superhydrophobic synergistic antibacterial surfaces, with emphasis on antibacterial mechanisms, wettability regulation strategies, and representative application fields. By integrating bioinspired micro/nanostructured design with interfacial superhydrophobicity, these surfaces achieve non-chemical, broad-spectrum, and long-lasting antibacterial performance through mechanisms such as inhibition of microbial adhesion, physical disruption of bacterial membranes, and self-cleaning effects. Furthermore, the application potential of such surfaces in medical devices, food packaging, marine and industrial anti-fouling, public facilities, water environments, functional textiles, and wearable electronics was comprehensively discussed. Despite these significant advances, several fundamental and engineering challenges still hinder large-scale implementation and long-term application.

### 5.1 Current challenges

#### 5.1.1 Incomplete understanding of structure–wettability–bacterial interaction mechanisms

The multi-scale coupling mechanisms among structural parameters (such as morphology, size, and spacing), superhydrophobic states, and interfacial mechanical effects (puncturing, stretching, and shearing) remain insufficiently understood. Notably, the bactericidal performance of micro/nanostructured surfaces exhibits obvious strain-dependent differences, which is closely related to the distinct cell wall architecture and mechanical properties of different bacterial species. The peptidoglycan layer thickness of bacterial cell walls directly regulates the mechano-bactericidal efficacy of nanostructures, and Gram-positive bacteria with thick peptidoglycan layers may significantly weaken the antibacterial effect of nanopillar structures, resulting in unstable and strain-selective antibacterial performance. Such interspecific bacterial differences further complicate the interfacial interaction mechanism between superhydrophobic surfaces and bacteria. Addressing these issues requires the integration of cross-scale simulations, interfacial mechanics modeling, and in situ dynamic characterization techniques to clarify the differential interaction rules between diverse bacterial strains and functional surfaces.

#### 5.1.2 Limited manufacturability, repeatability, and economic viability

Advanced micro/nano fabrication techniques achieve precise surface structuring but suffer from poor scalability, low throughput, and insufficient substrate compatibility. The sophisticated manufacturing processes and expensive raw materials further bring high production costs, which severely hinder commercial translation. Despite the growing superhydrophobic coating industry, excessive fabrication complexity and economic investment restrict large-scale application. Owing to the cost-performance mismatch in conventional scenarios, such high-performance bioinspired antibacterial surfaces are only economically viable for high-value niche applications with severe biofouling risks. In addition, many functional modifiers and fabrication reagents adopted in surface preparation, including fluorinated chemicals widely used for reducing surface energy, have inherent defects of poor environmental friendliness and potential biological hazards. The lack of systematic cost-benefit analysis balancing antibacterial functionality, biosafety and ecological risks further limits the practical industrial promotion of these surfaces. Therefore, low-cost, scalable, and repeatable manufacturing technologies with low biological and ecological toxicity are essential to break current limitations and realize practical industrial deployment.

#### 5.1.3 Insufficient stability under complex service environments

In environments involving moisture, high salinity, mechanical abrasion, or biological fluids, micro/nanostructures are susceptible to wear, structural degradation, or wettability transition. In addition, surface contamination and fouling may mask functional structures and weaken antibacterial performance. Beyond structural and wettability instability, the long-term service risks of functional surfaces are also prominent. For instance, antibacterial agents such as silver and copper nanoparticles commonly incorporated to strengthen bactericidal efficacy will undergo uncontrolled cation release during long-term service. The continuous leakage of metal ions and micro/nano particles not only causes cytotoxicity to mammalian cells and induces potential inflammatory responses in biomedical scenarios but also brings persistent ecotoxicological threats to the ecological environment. Therefore, constructing surfaces with enhanced mechanical durability, chemical stability, self-healing capability and controlled agent release performance is a critical challenge for future development.

#### 5.1.4 Limited multifunctional integration

Practical applications often require the simultaneous realization of multiple functions, such as antibacterial activity, anti-fouling behavior, corrosion resistance, oil repellency, and organic pollutant barriers. However, most current designs focus primarily on single-function antibacterial or adhesion-inhibiting performance. Moreover, the introduction of antibacterial functional components often conflicts with biocompatibility and environmental safety performance, making it difficult to balance high-efficiency bactericidal activity, biosafety and ecological friendliness. Achieving multifunctional integration through synergistic regulation of structure, wettability, and surface energy, while coordinating functional efficacy and safety performance, remains an important research direction.

#### 5.1.5 Limited data availability and intelligent design capability

The performance of micro/nanostructure–superhydrophobic antibacterial surfaces is governed by complex interactions among structural, wettability, and biological factors. Critically, there is a prominent lack of unified and standardized bactericidal evaluation protocols for functional surfaces. The absence of universal assay strategies and test standards leads to inconsistent test conditions, strain selection and evaluation indicators in different studies, which hinders horizontal comparison of research results and transformation of laboratory achievements. Meanwhile, the lack of standardized datasets covering different bacterial strains, service environments and biosafety performance further limits the application of artificial intelligence (AI) for surface design, performance prediction, and process optimization. Establishing unified evaluation specifications and reliable data resources to build AI-assisted design frameworks remains a key challenge.

#### 5.1.6 Insufficient evaluation of biosafety and long-term reliability

Although physical antibacterial strategies based on micro/nanostructures reduce reliance on chemical agents, they still have unresolved biosafety risks. On the one hand, the mechanical bactericidal structure may cause inadvertent damage to normal mammalian cells, and the cytotoxicity induced by uncontrolled release of metal antibacterial nanoparticles and toxic fluorinated modifiers will greatly weaken the application value of such surfaces in medical scenarios. On the other hand, existing evaluations mostly focus on short-term antibacterial efficacy, lacking systematic research on long-term biological toxicity, cell compatibility and ecological toxicological threats caused by particle and chemical leakage during the full service life. In addition, the long-term stability of antibacterial performance for different bacterial strains under continuous service conditions has not been systematically verified. Establishing comprehensive biosafety assessment protocols, long-term reliability testing standards and ecological risk evaluation systems is essential for clinical and industrial adoption.

### 5.2 Future prospects

The future development of micro/nanostructure–super-hydrophobic synergistic antibacterial technologies will rely on the deep integration of theoretical modeling, fabrication processes, advanced materials, and bioinspired strategies, moving toward intelligent, multifunctional, safe and engineering-oriented systems.

#### 5.2.1 Establish unified theoretical models of structure–wettability–interface mechanics

By combining multi-scale simulations, machine learning approaches, interfacial mechanics modeling, and *in situ* observation techniques, quantitative relationships among structural geometry, wettability states, and bacterial mechanical responses can be established. Focusing on the differential interaction mechanisms between different bacterial strains (Gram-positive and Gram-negative bacteria) and functional surfaces, the strain-adaptive design rules of micro/nanostructures can be clarified, enabling a transition from empirical design to predictive and programmable surface engineering.

#### 5.2.2 Develop scalable, high-throughput, and low-cost fabrication technologies

Combining high-speed laser processing, roll-to-roll nanoimprinting and light-field-assisted fabrication enables fast, uniform structuring on metals, polymers, ceramics and flexible substrates. Such low-cost, high-throughput composite processing is expected to cut manufacturing expenses and boost the industrial scaling of bioinspired antibacterial surfaces. Meanwhile, environmentally friendly and low-toxicity fabrication materials and modification strategies should be prioritized to replace harmful fluorinated reagents and uncontrollable metal nanoparticle systems, reducing production costs and inherent biological and ecological risks.

#### 5.2.3 Design highly durable, self-healing, and environmentally adaptive surfaces

Future antibacterial interfaces may incorporate hard coatings, composite materials, nano-reinforced architectures, self-healing polymers, and renewable wetting layers to withstand mechanical wear, chemical corrosion, biological fouling, and environmental fluctuations, ensuring long-term stability and maintainability. In particular, intelligent controlled-release functional systems should be developed to avoid excessive leakage of antibacterial components, thereby eliminating long-term cytotoxicity and ecological pollution risks, and improving the service safety of functional surfaces.

#### 5.2.4 Achieve multifunctional and coupled interface design

Through cross-scale structural engineering, gradient and Janus surface designs, multi-physical field coupling (including light, electric, magnetic, and thermal stimuli), and dynamically switchable wettability, multifunctional interfaces integrating antibacterial, anti-fouling, anti-corrosion, biocompatibility, and intelligent responsiveness can be realized to meet diverse application requirements. On the premise of ensuring high-efficiency and broad-spectrum antibacterial performance, the coordinated optimization of functional efficacy, cell compatibility and environmental friendliness should be realized to break the functional-safety contradiction of traditional surfaces.

#### 5.2.5 AI-assisted intelligent design and optimization

AI and machine learning are expected to accelerate the design and optimization of antibacterial surfaces by uncovering structure–property relationships and predicting performance. Combined with high-throughput experiments and computational modeling, AI can improve design efficiency, fabrication precision, and the development of multifunctional antibacterial interfaces. Based on unified test standards, a systematic performance database covering different bacterial strains, service environments and biosafety indicators should be constructed to provide reliable data support for intelligent design.

#### 5.2.6 Improve biosafety assessment and long-term service evaluation systems

A unified, standardized and comprehensive evaluation system for bactericidal performance and biosafety should be established to fill the methodological gaps in existing assessment schemes. More rigorous *in vitro* and *in vivo* evaluation frameworks need to be constructed, incorporating multi-strain bacterial models covering typical Gram-positive and Gram-negative bacteria, complex biological environments, cyclic lifetime testing, and realistic microbial community simulations. Furthermore, long-term cytotoxicity, tissue compatibility and ecotoxicological assessments throughout the service cycle should be added, and cost-benefit analysis balancing antibacterial function and biosafety should be carried out to fully support the safe application of such technologies in medicine, food safety, and marine engineering.

With continued advances in micro/nano fabrication, biointerface science, and multi-physics simulations, micro/nanostructure–superhydrophobic synergistic antibacterial technologies are expected to achieve a new generation of breakthroughs. Future research will progressively shift from experience-driven to model-driven design, from single-function optimization to multifunctional integration, and from laboratory validation to real-world application scenarios. Through interdisciplinary collaboration, the development of smarter, more durable, safer and environmentally sustainable antibacterial surfaces with broad engineering and medical value is anticipated.
